# The Role of Positive and Negative Information Processing in COVID-19 Vaccine Uptake in Women of Generation X, Y, and Z: The Power of Good is Stronger Than Bad in Youngsters?

**DOI:** 10.3389/fpsyg.2022.925675

**Published:** 2022-08-05

**Authors:** Eszter Eniko Marschalko, Kinga Szabo, Ibolya Kotta, Kinga Kalcza-Janosi

**Affiliations:** Applied Psychology Department, Faculty of Psychology and Educational Sciences, Babes-Bolyai University, Cluj-Napoca, Romania

**Keywords:** vaccine uptake, vaccine hesitancy, psychological flexibility, generational identity, health belief model, women, cognition, health

## Abstract

**Background:**

Positive and negative focus in information processing associated with age has a diverse role in COVID-19 vaccine uptake. The aim of the study was the exploration of the generational diversity among psychological predictors of COVID-19 vaccine uptake.

**Methods:**

A cross-sectional research was conducted. The sample included 978 Hungarian women. Based on former literature findings, the COVID-19 vaccine uptake predictors were chosen from the health beliefs model, COVID-19 vaccine hesitancy, and psychological flexibility. Multivariate logistic regression analysis was conducted to investigate the predictors of COVID-19 vaccine uptake in women of Gen X, Gen Y, and Gen Z.

**Results:**

In Gen X women, the influence of significant predictors are more prone to the positivity in COVID-19 vaccine uptake behavior, *perceived benefits* being the most relevant, increasing the likelihood of vaccine uptake more than four times. In Gen Y women, *perceived barriers, lack of confidence/skepticism* and *avoidance* significantly reduce the probability of vaccine uptake, showing an accentuated negative focus in information processing related to COVID-19 vaccination. The vaccine uptake in Gen Z is predicted only by the *perceived benefits*, and the likelihood of COVID-19 vaccine uptake is heightened in chance more than 19 times.

**Conclusion:**

Women belonging to Gen X or Gen Y, the *perceived benefits* hold the key to vaccine uptake, while in women of Gen Z, low risks, lack of threats, and accessibility could motivate the decision of vaccine uptake. The findings are useful in generation-adapted vaccination campaigns and can also serve as inspiration for evolutionary psychology studies on health behavior and the broad area of study in cognitive biases in health information processing.

## Introduction

The COVID-19 disease control is a still ongoing worldwide phenomenon in 2022. All global epidemiological waves of the disease, even the Omicron variant, were targeted effectively with the help of the COVID-19 vaccines, which significantly reduced the emergency cases and hospitalization risk of ill patients (Embi et al., 2021; Thompson et al., 2022). The government in many countries introduced the booster vaccine protocol, with the third and fourth dose (Falsey et al., 2021; Patalon et al., 2021), for lowering the risk of severe symptoms in their population. Despite the presence of scientifically significant data on the beneficial effects of the COVID-19 vaccines, the uptake intention of individuals globally is far from ideal, even if the vaccines are available.

### Age and Information Interpretation Bias in Health Behavior

Psychologically, individual illness prevention behavior is influenced by negative and positive information processing (Taylor et al., 2000; Baumeister et al., 2001). The role of different factors varies across domains of health behavior. In different areas of COVID-19 disease prevention with a medical priority (social distancing and hygiene) negative information processing factors (linked to health threats, e.g., infection prevention), whilst in other areas (information seeking and health behavior/ healthy lifestyle) positive information related seemed to count more (Marschalko et al., 2021). The vaccination intention and actual uptake decision were predicted in many COVID-19-related studies by an amalgam of psychological factors which have personal beliefs, attitudes, and cognitive evaluations as a consolidating base. These factors were targeted in former studies through comprehensive models, for example, the health belief model, in the context of intra-individual variables which favor optimism and appreciation of personal resources, for example, psychological flexibility, and also in vaccine-specific approaches, for example, vaccine hesitancy.

Age is a positive predictor of COVID-19 vaccine uptake (Bish et al., 2011) and older individuals tend to listen more to the physician's vaccine recommendations (Coe et al., 2012; Shmueli, 2020; Wong et al., 2020; Hossain et al., 2021). Furthermore, older individuals tend to favor positive over negative information, a preferential shift toward emotionally positive information was highlighted in the literature (Carstensen and Mikels, 2005; Carstensen, 2006; Reed et al., 2014). In case of elderly individuals, the underlying mechanisms were linked especially to better emotional regulation skills (Kensinger and Schacter, 2008; Leclerc and Kensinger, 2008; Brassen et al., 2011) and to a more adapted assessment of reality. Due to the passing of time, older people perceive the positive side of personal circumstances and interpret happenings in the social and emotional contexts in a more positive way (Carstensen and Mikels, 2005; Carstensen, 2006).

A quantitative meta-analytic study, that included 100 studies and more than 7,000 participants, concluded that the negativity bias is more likely in youth (Reed et al., 2014). The results are consistent with evolutionary-focused findings (Baumeister et al., 2001). The age-related positivity effect on cognition is highlighted in many studies (Isaacowitz and Blanchard-Fields, 2012; Chowdhury et al., 2013). Optimism, as the tendency to overestimate future positive events over negative ones (Weinstein, 1980; Chowdhury et al., 2013), however, goes against this age-related progressive positivity effect, as it was evinced in younger individuals, as well (Isaacowitz, 2005; Lachman et al., 2008). The explanation of the presence of optimism in young adults was linked to age-related brain development processes, which favor the positively biased assessment of desirable outcomes (Sharot et al., 2011, 2012a,b). Reed et al. (2014) argue that behavior, cognition, and emotion, potentially holding a bias risk, are influenced by personal motivation. Murphy and Isaacowitz (2008) argued that in the case of emotional stimuli, there were rather a few age-related differences in positive and negative interpretation if results were compared to neutral stimuli, and smaller effects were found for emotion salience and negativity preferences in older individuals compared to younger adults.

### The Role of the Health Belief Model in COVID-19 Vaccine Uptake Prediction

In an integrated framework on general and specific health behavior, the *health belief model (HBM)* includes a variety of positive and negative factors which can contribute to personal decisions. HBM states that general prevention and health maintenance behavior is influenced by individual beliefs and benefits/risk assessments in which the personal cognition processing is conclusive for action. The model presents the following factors: *perceived susceptibility, perceived severity, perceived benefits, perceived barriers, cues to action, and self-efficacy* (Rosenstock, 1974; Champion and Skinner, 2008; Orji et al., 2012). The HBM suggests that individual characteristics of a patient (e.g., demographics and knowledge) directly impacts individual beliefs and lead to individual intentions and health behavior decisions. The HBM model was highlighted as an important role in vaccine uptake in the case of the H1N1 Influenza vaccine (Bish et al., 2011; Coe et al., 2012), Swine Flu vaccine (Myers and Goodwin, 2011), Hepatitis B vaccine (Huynh et al., 2021), and COVID-19 vaccines (Mercadante and Law, 2020; Shmueli, 2020; Wong et al., 2020; Hossain et al., 2021; Zampetakis and Melas, 2021). A systematic review pointed toward the important role of HBM in the case of general vaccine uptake in adults with a high-risk physical health condition (Borthwick et al., 2020). Perceived benefits, along with perceived barriers, were evinced having a significant role in the vaccine uptake decision of individuals (Myers and Goodwin, 2011; Mercadante and Law, 2020; Shmueli, 2020; Wong et al., 2020; Hossain et al., 2021). Risk perception or susceptibility influenced the vaccination intent (Bish et al., 2011; Coe et al., 2012; Shmueli, 2020; Wong et al., 2020; Hossain et al., 2021; Zampetakis and Melas, 2021). Past flu vaccine uptake was predictive of new vaccine uptake, highlighting a general beneficial attitude toward vaccines as a method of prevention of illnesses (Bish et al., 2011; Myers and Goodwin, 2011; Coe et al., 2012).

### The Role of Vaccine Hesitancy in COVID-19 Vaccine Uptake Prediction

In vaccine-specific approach, the *vaccine hesitancy (VH)* is defined on a behavior continuum in the literature, which comprises the possibility of total refusal of vaccine intake on one side and the acceptance of vaccine intake on the other. If the hesitancy is very strong then the uptake of the jab is refused by the patients (Dubé et al., 2013). The COVID-19 vaccine hesitancy was linked to the possibility of vaccine conspiracy beliefs (Freeman et al., 2020), lack of confidence in beneficial effects, and vaccine risk appreciation (Rodriguez et al., 2021). Vaccine hesitancy is also defined by skepticism, vaccine risk, and fear of the COVID-19 vaccine (Kotta et al., 2021a). Many recent studies evinced the significant role of hesitancy on COVID-19 vaccine uptake (Bhopal and Nielsen, 2020; Lucia et al., 2020; Machingaidze and Wiysonge, 2021; Solís Arce et al., 2021). Demographic variables also play their role in VH, and often older, well-educated individuals or those who suffer from chronic diseases are more open to accepting the vaccines (Freeman et al., 2020; Al Janabi and Pino, 2021; Al-Mohaithef et al., 2021; Paul et al., 2021; Truong et al., 2021).

### The Role of Psychological Flexibility in COVID-19 Vaccine Uptake Prediction

From an intra-individual perspective, an important role in vaccine uptake is played by *psychological flexibility* (Wang and Zhang, 2021). This variable is defined through the individual's ability to accept rather than avoid negative thoughts and emotions about life circumstances (Hayes et al., 2006). Psychologically flexible individuals feel less anxiety and can cope resiliently in ambiguous circumstances even in health-related contexts. Individuals with chronic respiratory disease with higher reported levels of psychological flexibility were more likely to receive the seasonal influenza vaccination (Cheung and Mak, 2016). Psychologically more flexible parents tend to see the beneficial effects of COVID-19 vaccines in the case of their children (Wong et al., 2021). Psychological flexibility favors lifestyle-related prevention behavior in the COVID-19 pandemic (e.g., healthy diet and weekly exercise) and tends to have a stronger influence in the case of younger generations (Kotta et al., 2021b; Marschalko et al., 2021).

### Aim of the Study

COVID-19 variants are continuously raising concerns in some parts of the world. COVID-19 vaccines will be necessary annually in some segments of the population. The personal cognitive interpretation tendencies (e.g., positive and negative bias/ focus), which guide health behavior and decisions like vaccine uptake, are diversely augmented in older and younger individuals. Based on results highlighted in the literature on positive and negative cognitive bias and shift in information processing associated with age, we assume a higher impact on COVID-19 vaccine uptake of benefits and positive interpretation-related variables in older individuals (Isaacowitz and Blanchard-Fields, 2012; Chowdhury et al., 2013) and a higher role of negative information processing-related variables in younger adults (Baumeister et al., 2001; Reed et al., 2014).

The aim of the study was the exploration of the generational diversity among psychological predictors of COVID-19 vaccine uptake. Considering the predictive role of the health-related beliefs (e.g., susceptibility, severity, benefits, barriers, cues to action), psychological flexibility (avoidance, acceptance, harnessing), COVID-19 vaccine hesitancy (skepticism, risk perception, fear) on vaccine uptake, as formerly highlighted in the literature, and the age-related vulnerability, the present study proposed the analysis of these variables in Gen Z, Gen Y, and Gen X. The differential predictive weight of these psychological variables at different ages can bring new insight to the literature.

## Measurement and Methods

### Participants

The sample was recruited from the general population of Hungary and Ethnic Hungarians in Romania (Transylvania), and the participants were Hungarian speakers. The snowball sampling method was used online, and the gathered participants included <15% males. For generalizability error avoidance purposes, the authors decided on the inclusion of female participants only. A total of 978 women were included in the study, and the authors grouped the participants into three distinct categories using a generation criteria list presented in the Dimock (2019) and the Beresford Research (n.d.) studies. A generation is a group of people born around the same time with similar characteristics, preferences, and values over their lifetimes: Gen Z (born 1997–2012, ages 10–25 years), Gen Y or Millennials (born 1981–1996, ages 26–41 years), and Gen X (born 1965–1980, ages 42–57 years). In the present study, the Gen X age interval was expanded (ages 42–64) so that the three examined generation sample size is approximately the same. Descriptive statistics of the participants are presented in Table 1, separately for the three generations (Gen Z, Gen Y, and Gen X).

**Table 1:** Baseline characteristics of the participants (*N* = 978).

	**Gen Z**	**Gen Y**	**Gen X**
	**(*n* = 227)**	**(*n* = 363)**	**(*n* = 388)**
**Age**	21.31 ± 1.85	34.92 ± 4.88	49.62 ± 5.21
**Education (*****n***, ***%*****)**			
8 grades or less	-	1 (0.3%)	-
Professional school/10 grades	1 (0.4%)	1 (0.3%)	5 (1.3%)
High school without baccalaureate	1 (0.4%)	4 (1.1%)	18 (4.6%)
Baccalaureate	124 (54.6%)	67 (18.2%)	84 (21.6%)
College, university	81 (35.7%)	163 (44.9%)	183 (47.2%)
Master degree	20 (8.8%)	114 (31.4%)	76 (19.6%)
Doctoral degree	-	11 (3.0%)	17 (4.4%)
Other	-	3 (0.8%)	5 (1.3%)
**Country (*****n***, ***%*****)**			
Ro	191 (84.1%)	98 (27%)	60 (15.5%)
Hu	36 (15.9%)	265 (73%)	328 (84.5%)
**Chronic disease (*****n***, ***%*****)**			
No	197 (86.8%)	291 (80.2%)	268 (69.1%)
Yes	30 (13.2%)	72 (19.8%)	120 (30.9%)
**BMI**	21.93 ± 4.01	24.31 ± 5.09	26.41 ± 5.29
**Diagnosed_COVID-19 (** ***n, %*** **)**			
No	155 (68.3%)	267 (73.6%)	282 (72.7%)
Yes	36 (15.9%)	58 (16%)	79 (20.4%)
Not sure	36 (15.9%)	38 (10.5%)	27 (7%)
**Flu vaccine past (** ***n, %*** **)**			
No	165 (72.7%)	301 (82.9%)	301 (77.6%)
Yes	62 (27.3%)	62 (17.1%)	87 (22.4%)
Susceptibility	3.16 ± 1.09	3.11 ± 1.17	2.68 ± 1.04
Severity	3.54 ± 1.11	3.68 ± 1.15	3.60 ± 1.22
Benefits	3.35 ± 1.39	3.26 ± 1.42	3.15 ± 1.51
Barriers	2.58 ± 1.06	2.63 ± 1.12	2.66 ± 1.15
Cues to action	3.27 ± 1.64	3.51 ± 1.79	3.68 ± 1.92
Avoidance	4.93 ± 1.35	5.25 ± 1.46	5.47 ± 1.44
Acceptance	4.73 ± 1.14	4.65 ± 1.24	4.64 ± 1.26
Harnessing	3.73 ± 1.12	3.34 ± 1.29	3.33 ± 1.29
Skepticism	2.79 ± 1.37	2.88 ± 1.42	2.97 ± 1.52
Risk	2.64 ± 1.10	2.65 ± 1.11	2.80 ± 1.25
Fear	1.49 ± 0.91	1.54 ± 0.95	1.59 ± 1.12

### Measurements

#### Demographic Information and COVID-19-Related Variables

A structured online questionnaire was elaborated to measure basic demographic information (age, country, and education), health-related variables (chronic disease, BMI, and flu vaccine past), and COVID-19-related variables (former or present COVID-19 diagnosis and vaccine uptake). The vaccine uptake was divided into two categories (not vaccinated and vaccinated).

#### Health Belief Model

The following constructs of the HBM model were measured: *perceived susceptibility* (subjective assessment of the risk of developing a health problem, e.g., “I am at risk of getting COVID-19”), *perceived severity* (subjective assessment of the severity of a health problem and its potential consequences, e.g., “I believe that COVID-19 is a severe health problem”), *perceived benefits* (individual and community benefits of taking action, e.g., “COVID-19 vaccines will work in preventing the disease”), *perceived barriers* (safety and cost concerns of taking action, e.g., “Not enough research done on COVID-19 vaccines”), and *cues to action* (a trigger, an internal or external cue that is necessary for promoting engagement in health-promoting behaviors, e.g., “Family or close friend tested positive for COVID-19”). The context-specific/situational HBM items related to the exposure to COVID-19 were elaborated by Chu and Liu (2021). The Cronbach's alpha values in this study were as follows:0.89 for susceptibility, 0.91 for severity, 0.97 for benefits, 0.86 for barriers, and 0.66 for cues to action.

**Multidimensional COVID-19 Vaccine Hesitancy Scale (CoVaH)** is a 15-item self-report measure elaborated by Kotta et al. (2021a). The scale assesses the beliefs and attitudes beneath vaccination hesitancy and reasons for vaccine refusal in the context of COVID-19 through three subscales: *vaccine risk* [e.g., “COVID-19 vaccines can lead to severe allergic reactions (anaphylactic shock)”] measures the hesitancy due to possible adverse effects of the vaccines, *fear* [e.g., “I have chills (goosebumps) when I think about being vaccinated with one of the COVID-19 vaccines”] reflects the individual emotional and physiological reactions related to being vaccinated, and *lack of confidence/skepticism* [e.g., “COVID-19 vaccines are effective (R)”] is the hesitancy due to lack of confidence in the vaccine's beneficial effect on health and community. The scale was shown to have very good psychometric properties, Cronbach's alpha values were α = 0.94 for skepticism, α = 0.89 for risk, and α = 0.89 for fear subscales, while the internal validity of the total scale is also excellent α = 0.94 (Kotta et al., 2021a). In this study, Cronbach's alpha values were 0.95, 0.90, and 0.90 for skepticism, risk, and fear, respectively.

#### COVID-19 Health-Related Personal Psychological Flexibility Index (PPFI)

The 15 items of the Personal Psychological Flexibility Index (Kashdan et al., 2020) were used for measuring the trait-like ability to pursue valued life aims and daily goals despite the presence of distress. In the present research, a COVID-19 pandemic and health-related distress were targeted, and therefore the scale instruction was reformulated accordingly: “*Please take a few moments to think of an important goal that you are working on related to your health maintenance during COVID-19 pandemic. It must be one and only one goal. Don't choose too quickly. Take a few moments to think about it. After you choose the goal, please write it in the following blank: __. For each statement below, select the rating that best describes your thoughts and feelings about this goal*.” The PPFI targets flexibility on three subscales: acceptance (e.g., “I accept the setbacks when pursuing this goal”), avoidance (e.g., “I avoid the most difficult goal-related tasks”), and harnessing (e.g., “When faced with obstacles related to this goal, my frustration serves to energize me”). A 7-point Likert scale was applied for recording the answers from strongly disagree to strongly agree. The alpha coefficient of the total scale was 0.84, while test-retest reliability was also appropriate (Kashdan et al., 2020). In this study, Cronbach's alpha was 0.75, 0.88, and 0.72 for acceptance, avoidance, and harnessing, respectively.

### Procedure

A cross-sectional study was carried out between May and June 2021, a year after the outbreak of the pandemic, when mass vaccination had already became available for almost everyone in Europe. A convenience sampling method, namely the snowball technique was applied; the online survey was promoted on social media platforms. After confirming eligibility (18 years of age or over) and providing informed consent to participate in the study, respondents completed the survey on Google Forms containing the demographic, health, and COVID-19-related queries and the COVID-19 Health-Related Personal Psychological Flexibility Index, the HBM Scale, and the CoVaH *Scale*. Anonymity was assured, and no personal identifiers were provided. Survey completion took ~15–20 min.

### Data Screening

The online sampling method provided <15% male participants, and the authors decided upon a woman-focused analysis and data interpretations in a gender-specific manner, to lower the chance of bias in the generalizability of results. To investigate the established relations, SPSS (Statistical Package for the Social Sciences) version 23.0 was performed. The first set of analyses included screening data based on Field's (2009) and Tabachnick and Fidell's (2019) work. There were no variables with 5% or more missing values. Standardized *z*-scores were created for the major continuous variables to assess the outliers. There were 95% of cases with an absolute value <1.96, and none of the cases had a value higher than 3.29. Due to the large sample size (*N* = 978), the normality distribution was checked using visual analysis and it revealed a mostly normal distributed sample.

### Data Analysis

For statistically appropriate sample size calculation, a priori power analysis was performed using G^*^Power3 (Faul et al., 2007). All the data were presented as mean (M) and standard deviation (SD) for continuous variables and frequencies/percentages for categorical variables (see Table 1). The internal consistency of scales and subscales was assessed by calculating Cronbach alpha's reliability values. The probability value was set at 0.05. Three multivariate binary logistic regression analyses were conducted on three different generational groups (Gen X, Gen Y, and Gen Z) to establish the predictors of vaccine uptake. These predictors were chosen based on the literature. The assumptions were tested and the data fit the regression model. In the regression models, categorical variables were introduced as dummy variables and the unstandardized regression coefficients (*B*), standard errors (*SE*), WALD statistics, odds ratio [Exp (B)], and Nagelkerke *R*^2^ value were calculated.

## Results

**Figure 1:**
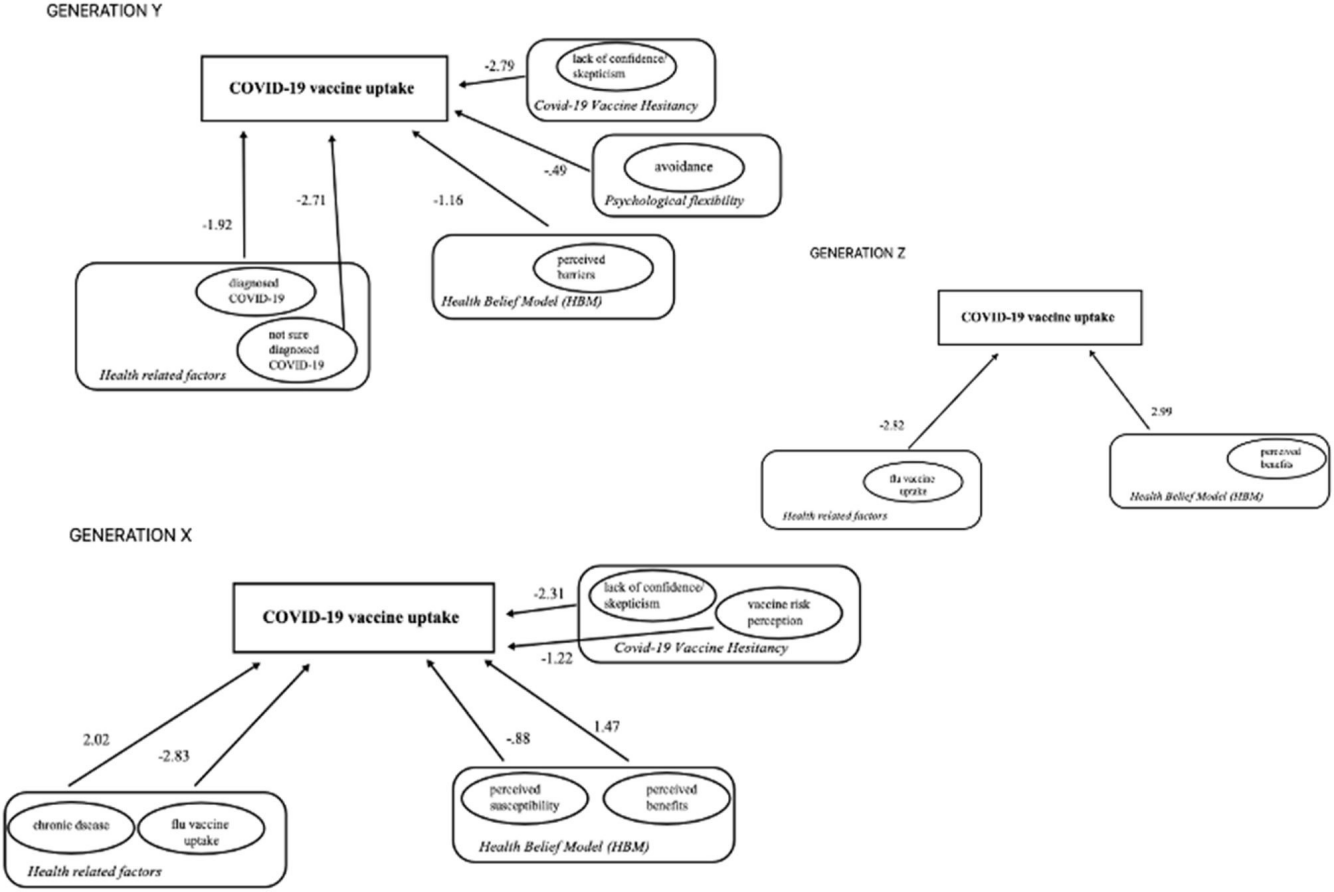
Predictors of COVID-19 vaccine uptake in women of Gen X, Y, and Z. Values represent unstandardized beta (B) values from multivariate binary logistic regression results.

To investigate the generational diversity among psychological predictors of COVID-19 vaccine uptake, three logistic regression models were calculated for each generation. The binary outcome variable of the predictor model was the participant's COVID-19 vaccine uptake, in the following way: (1) *not vaccinated* and (2) *vaccinated*. Based on the theoretical background of the study that proves the importance of the health-related variables (e.g., flu vaccination or having a chronic disease) of the HBM model, psychological flexibility, and the vaccine hesitancy in predicting vaccination, no presumption on the differential importance was considered beforehand in the predictor analysis. Therefore, the enter method of the regression analysis was chosen, which is the most recommended method for building theories. The enter method is a forced entry method, where all the input variables are included simultaneously. This was considered by the authors to be the most suitable choice because all the predictors were given equal importance in this explorative research. Age and gender were not included in the analysis, because the generation grouping was made on age intervals, and there were only women participants included in this research, with similar ethnical backgrounds. Table 2 and Figure 1 present the results of the multivariate binary logistic regression.

**Table 2:** Multivariate binary logistic regression results on COVID-19 vaccine uptake in women of Gen Z, Gen Y, and Gen X.

**Predictor**	**Gen Z**					**Gen Y**					**Gen X**				
	**(*****n*** **=** **227)**					**(*****n*** **=** **363)**					**(*****n*** **=** **388)**				
	**B**	**S.E. B**	**Wald**	**Exp (B)**	**CI (95%)**	**B**	**S.E. B**	**Wald**	**Exp (B)**	**CI (95%)**	**B**	**S.E. B**	**Wald**	**Exp (B)**	**CI (95%)**
(Constant)	−6.80	9.38	0.53	0.01		20.75	6.19	11.26	1030651243.00		16.11	5.19	9.65	9925714.79	
Chronic disease (no = 0, yes = 1)	−0.44	1.34	0.11	0.64	0.05, 8.85	−1.27	0.78	2.66	0.28	0.06, 1.29	2.02	0.87	5.42^**^	7.56	1.38, 41.50
BMI	0.26	0.14	3.43	1.30	0.99, 1.72	−0.02	0.06	0.08	0.98	0.87, 1.11	0.04	0.06	0.50	1.04	0.93, 1.17
Diagnosed COVID-19 Yes	1.20	1.25	0.90	3.28	0.28, 78.00	−1.92	0.78	6.07^**^	0.15	0.03,0.68	0.28	1.15	0.06	1.32	0.14, 12.69
Not sure	0.24	1.42	0.03	1.27	0.08, 20.44	−2.71	1.11	5.98^**^	0.07	0.01,0.58	−2.51	1.54	2.66	0.08	0.01,0.1.66
Flu vaccine uptake (in the past) (no = 0, yes = 1)	−2.82	1.18	5.73^**^	0.06	0.01,0.60	−1.91	1.03	3.41	0.15	0.02, 1.12	−2.83	1.40	4.14^**^	0.06	0.01, 0.90
Perceived susceptibility	0.11	0.53	0.04	1.11	0.39, 3.17	−0.48	0.29	2.71	0.62	0.35, 1.10	−0.88	0.38	5.37^**^	0.41	0.20, 0.87
Perceived severity	−0.63	0.51	1.56	0.53	0.20, 1.44	0.52	0.33	2.49	1.69	0.88, 3.23	−0.43	0.40	1.12	0.65	0.30, 1.44
Perceived benefits	2.99	1.29	5.21^**^	19.20	1.52, 242.55	0.06	0.55	0.01	1.06	0.36, 3.14	1.47	0.66	4.90^**^	4.33	1.18, 15.85
Perceived barriers	−0.75	0.74	1.03	0.47	0.11, 2.02	−1.16	0.41	8.02^**^	0.31	0.14,0.70	−0.34	0.54	0.39	0.71	0.25, 2.06
Cues to action	−0.04	0.34	0.01	0.96	0.49, 1.88	0.02	0.19	0.01	1.02	0.70, 1.47	0.03	0.23	0.02	1.03	0.66, 1.61
Avoidance	−0.43	0.36	1.45	0.65	0.32, 1.31	−0.49	0.22	4.72^**^	0.61	0.39,0.95	−0.20	0.26	0.62	0.82	0.50, 1.35
Acceptance	0.72	0.57	1.63	2.06	0.68, 6.22	−0.06	0.25	0.05	0.94	0.58, 1.54	−0.31	0.27	1.31	0.73	0.43, 1.25
Harnessing	0.23	0.40	0.32	1.25	0.57, 2.75	−0.24	0.22	1.19	0.79	0.51,1.21	0.11	0.26	0.20	1.12	0.68, 1.86
Lack of confidence/ skepticism	−0.61	0.95	0.42	0.54	0.08, 3.50	−2.79	0.67	17.17^***^	0.06	0.02,0.23	−2.31	0.65	12.68^***^	0.10	0.03,0.35
Vaccine risk perception	−0.01	0.69	0.01	1.00	0.25, 3.85	0.67	0.44	2.36	1.96	0.83, 4.64	−1.22	0.48	6.44^**^	0.30	0.11,0.76
Fear of vaccine	−1.37	0.83	2.74	0.25	0.05, 1.29	−0.73	0.41	3.25	0.48	0.22, 1.06	0.05	0.30	0.03	1.05	0.58, 1.91
Nagelkerke *R^2^*	0.91					0.88					0.91				

*** *p < 0.001*,

** *p < 0.01. We examined the predictor role of Health belief model (perceived severity, perceived benefits, perceived barriers, cues to action), Psychological flexibility (avoidance, acceptance, harnessing), and COVID-19 Vaccine hesitancy (vaccine risk/skepticism, vaccine risk perception, fear of vaccine) on COVID-19 vaccine uptake, in the women of Gen Z, Y, and X*.

*Statistically significant predictors are presented with bold style and accompanied by stars*.

The logistic regression model included as predictor variables, the following: the participants' education level, health behavior-related factors (chronic disease status, BMI value, being diagnosed or not with this disease, getting other flu vaccines in the past), the factors of vaccine hesitancy, the components of the HBM model, and the psychological flexibility.

The analyzed model for Gen Z explained 91% (Nagelkerke *R*^2^) of the variance in COVID-19 vaccine uptake. Of all the psychological predictors, only the *perceived benefits (HBM)* were associated with the increased likelihood of the vaccine uptake. In health behavior-related variables, past flu vaccine uptake was a negative predictor of COVID-19 vaccine uptake chance in this generation (see Table 2 and Figure 1).

Gen Y had many significant predictors of COVID-19 vaccine uptake. The model explained 88% (Nagelkerke *R*^2^) of the variance of this behavior. *Perceived barriers* (HBM), *avoidance* (psychological flexibility), and *lack of confidence/skepticism* (CoVaH) in COVID-19 vaccine beneficial effects lowered the probability of getting the vaccine. As a health behavior-related predictor, being diagnosed with COVID-19 disease in Gen X women is associated with a reduction in the likelihood of COVID-19 vaccine uptake behavior. None of the analyzed predictors contoured as positive predictors in this case.

In the case of the oldest generation of women, Gen X, the model explained 91% (Nagelkerke *R*^2^) of the variance of COVID-19 vaccine uptake. From the analyzed psychological predictors, the *perceived susceptibility* (HBM) and *perceived benefits* (HBM) played an important positive role in increasing the chance of COVID-19 vaccine uptake behavior. On the other hand, the COVID-19 vaccine hesitancy-related variables, like *lack of confidence/skepticism* in the vaccine's beneficial effect and the *vaccine risk* perception were significantly associated with a reduction in the likelihood of COVID-19 vaccine uptake. Health behavior-related predictors also hold an important role in this generation. The participants' chronic disease, the actual COVID-19 disease diagnosis, had a positive impact on the likelihood of COVID-19 vaccine uptake, and the seasonal/past flu vaccine uptake played a negative role in the chance of COVID-19 vaccine uptake behavior.

## Discussion and Conclusion

COVID-19 disease control and prevention is efficiently targeted with vaccination. The newest variants, like Omicron, were targeted with booster dose application (Embi et al., 2021; Thompson et al., 2022), and there is a high chance of implementing COVID-19 vaccines in prevention schedules, similarly to the seasonal flu management. The present study was motivated by the scarcity of literature on generational diversity related to COVID-19 vaccine uptake, in the context of psychological predictors related to HBM, COVID-19 vaccine hesitancy, and psychological flexibility. The chosen variables were interpreted in the context of positive and negative information processing preferences associating age, in three generations of women: Gen X, Gen Y, and Gen Z.

### The Role of Demographic and Health-Related Variables on COVID-19 Vaccine Uptake

From the assessed demographic and individually relevant variables (education, BMI), none contoured in a statistically significant way.

The only health behavior-related predictor which was important in at least two generations of women (Gen Z, Gen X) was the previous flu vaccine uptake. In both cases, this is a significant negative predictor of the likelihood of COVID-19 vaccine uptake. This result is in contradiction with former results in the literature, which have shown a positive association between past flu vaccine uptake and new vaccine uptake (Bish et al., 2011; Myers and Goodwin, 2011; Coe et al., 2012).

The presence of chronic disease was a significant predictor of vaccine uptake only in the case of Gen X, making the chance of COVID-19 vaccination higher than seven times. The result is in line with other findings on chronic disease and vaccine uptake (Freeman et al., 2020; Al Janabi and Pino, 2021; Al-Mohaithef et al., 2021; Paul et al., 2021; Truong et al., 2021), but none of these studies focused on generational diversity. Further studies are needed to analyze the potential moderator role of generational identity on the relationship between chronic disease and vaccination uptake.

The actual COVID-19 infection and related consequences had a diverse role in predicting the likelihood of COVID-19 vaccine uptake in two generations. Interestingly, in the case of Gen Y, the infection with the coronavirus made the vaccine uptake less likely.

### Health Belief Model and COVID-19 Vaccine Uptake

The results of the study show a significant diversity especially in the case of perceived benefits from HBM, which is the strongest predictor in Gen Z and Gen X in actual COVID-19 vaccine uptake decision. The perception of benefits raises the chance of getting vaccinated more than 19 times in the case of Gen Z and more than 4 times in the case of Gen X. This variable from HBM is the strongest in both cases in the context of all considered psychological predictors, showing the important role of positive information processing-related aspects in COVID-19 vaccine uptake behavior. A new insight on the topic is related to the marked weight of positive information linked to the benefits of the vaccine in youngsters.

In the case of Gen X women, an important feature is the role of perceived susceptibility, and it is lowering the odds of getting the COVID-19 vaccine by 0.4 chance. Perceived barriers played a significant role only in the women of Gen Y, making it less probable for getting the jab with a 0.3 odds ratio.

The results on the HBM predictor role in COVID-19 vaccine uptake of different generations give partial support to the literature on the positive shift in information processing in the case of older adults (Carstensen, 2006; Isaacowitz and Blanchard-Fields, 2012; Chowdhury et al., 2013; Reed et al., 2014). The presence of such an important predictive power of perceived benefits in the case of individuals in their early twenties (Gen Z) is an intriguing result, because there is a scarcity of explanations and also of similar results in health psychology. Former studies indicate mostly the presence of negative information processing power over positive ones in young people (Baumeister et al., 2001; Reed et al., 2014), which in our case was present only in the case of Gen Y (age above 26). One possible explanation of the high power of benefit perception in the COVID-19 vaccine uptake in Gen Z can be linked to brain developmental phases in young adults, which may trigger optimism around future estimations of desirable outcomes (Sharot et al., 2011, 2012a,b). The benefits promised by vaccines linked to restrictions of COVID-19 lockdown being potentially abolished could have triggered motivationally the youngest of the participants in favor of positive perception and the usage of extensively positive bias in health-related decisions, like vaccine uptake. The rare context of COVID-19 lockdown probably could trigger the future time-limited perspective approach even in the youngest, activating the positivity bias effects in information processing. The relationship between limited time perspective and positivity was found in former studies (Henry et al., 2017). Erbey et al. (2020) highlighted the role of a complex interplay of psychosocial and emotional features in positivity effects in information interpretation, evidencing limited future time perspective with a significant role even in young participants. In this context, we can argue that if specific health-related situation puts at risk the individually motivating environments, and if the young adult faces situations in which he/she perceives his or her future time (life) as being limited, positive bias is likely to appear, in concordance with the social-emotionality theory, which was formerly highlighted in case of the life-span theory (Carstensen and Mikels, 2005; Carstensen, 2006).

In COVID-19 prevention behavior, the perceived benefits were highlighted in many studies (Myers and Goodwin, 2011; Coe et al., 2012; Mercadante and Law, 2020; Shmueli, 2020; Wong et al., 2020; Hossain et al., 2021), but the literature is scarce on age and generational identity-related results. Generational diversity was shown in COVID-19 prevention behavior (Kotta et al., 2021b; Marschalko et al., 2021), but there is a high need for further understanding of this phenomenon.

### Vaccine Hesitancy and COVID-19 Vaccine Uptake

COVID-19 vaccine hesitancy variables, such as skepticism, risk perception (on adverse effects), and fear contours only in two cases in COVID-19 vaccine uptake prediction, namely in Generation Y and Generation X. In the case of Gen Y, lack of confidence/skepticism lowered the chance of getting vaccinated by.06 times. In the case of Gen X, lack of confidence/skepticism and vaccine risk perception linked to COVID-19 vaccines lowered the COVID-19 vaccine uptake chance by 0.10 times. No predictor related to COVID-19 vaccine hesitancy was evidenced in the case of Gen Z women, and this data are pinpointing rather a lack of hesitancy in the women of the youngest generation.

### Psychological Flexibility and COVID-19 Vaccine Uptake

Our findings on psychological flexibility highlighted only one predictor related to this variable, namely in the case of Gen Y women, only the avoidance contoured as a significant negative predictor of COVID-19 vaccine uptake behavior. This variable lowers the chance of COVID-19 vaccine uptake by .06 times. Our study failed to show the results on the positive role of psychological flexibility on health behaviors and vaccine uptake, as in former studies (Cheung and Mak, 2016; Kotta et al., 2021b; Marschalko et al., 2021).

### The Role of Information Interpretation Bias in COVID-19 Vaccination of Women Belonging to Different Generations

#### Generation Z

Gathering all significant predictors in every analyzed generation of women, we can say that the most pronounced focus is on the benefits of the COVID-19 vaccine, and positivity focus shows up in the case of the adults up to 25 years (Gen Z). In youngsters, besides the seasonal flu vaccine uptake (negative predictor), only the perceived benefits count positively in the likelihood of COVID-19 vaccine uptake, raising the odds more than 19 times. The role of positive information (e.g. benefits) linked to benefits from HBM is pointing toward an extremely positive shift in health-related perceptions, cognitions, and emotions in the youngsters (see Figure 1). No vaccine hesitancy variable was highlighted significantly in this generation. The extensive positivity can be justified in the context of age-related brain development aspects, which favor optimism (Sharot et al., 2012a,b). This finding on the exclusive role of positive information in Gen Z's COVID-19 vaccine uptake needs further research because it could hold information on specific health-related circumstances in which evolutionary gains are reinterpreted by young individuals, and negative information interpretation could be reframed from “bad is stronger than good” (Baumeister et al., 2001) in the context of perceived limited future time and individual approaches (Henry et al., 2017; Erbey et al., 2020) into “good is much better than bad, if my time is limited.” The possible moderation effect of extreme lockdown could be in focus in this specific case and further studies are needed for the clarification of this new insight.

#### Generation Y

In the case of Gen Y, the chance of COVID-19 vaccine uptake is controlled mostly from a negative perspective. Those who got the infection tended to refuse the vaccine. Perceived barriers also played a role in lowering the probability of COVID-19 vaccine uptake behavior. Lack of confidence/skepticism in the beneficial effects of the COVID-19 vaccine lowered significantly the probability of COVID-19 vaccine uptake (see Figure 1). Every significant predictor contoured as a negative one for COVID-19 vaccine uptake in the case of women in the 26–42 years age categories (Gen Y). Even in the case of psychological flexibility, the only significant variable was related to avoidance and held a negative role in the likelihood of COVID-19 vaccine uptake. In the case of Gen Y women, an extended negative information process and focus were more present in general. Evolutionary gains (e.g. adaptation) are served with this negative focus (Baumeister et al., 2001; Reed et al., 2014), and in the case of older adults, in their middle adulthood, these are shown in our study as well (Reed et al., 2014).

#### Generation X

The predictors of COVID-19 vaccine uptake in Gen X are a mixture of positive and negative information processing-focused variables (see Figure 1). In this case, chronic disease is more likely, and it did hold a significant positive role in the uptake decision, heightening its chance more than seven times. From the analyzed significant psychological predictors, the perceived susceptibility (negatively) and the perceived benefits (positively) predicted the likelihood of the COVID-19 vaccine. Furthermore, the lack of confidence in the benefits and the vaccine risk perception of COVID-19 vaccine benefits both predict negatively the COVID-19 vaccine uptake behavior. Evaluating the weight of each predictor in the total regression model, we can say that, the positive predictor of perceived benefits is the most relevant, increasing the chance of actual vaccine uptake more than four times. In the case of Gen X women, the influence of significant predictors is more prone to positive information processing and positivity effect on cognition. The positive focus on the information processing of older adults was highlighted before in the literature, being backed up also by social-emotional theory (Carstensen and Mikels, 2005; Carstensen, 2006). Even if the risk is perceived and helps in health behavior adjustment (Marschalko et al., 2021), most of the time, the cognition and health behavior in older adults are influenced by positivity (Weinstein, 1980; Isaacowitz and Blanchard-Fields, 2012; Chowdhury et al., 2013; Reed et al., 2014). The present studies' positivity findings can also point toward the presence of better emotional regulation skills, which favors optimism (Brassen et al., 2011; Erbey et al., 2020). The presence of chronic disease can be interpreted in this case also in the context of personal remaining time or limited future time, which posits a higher emphasis on positive assessment and on emotionally and socially relevant and positive aspects (Carstensen, 2006) in which a COVID-19 vaccine potentially can bring benefits.

Taking all psychological predictors into account, it can be concluded that there is significant diversity across generations Z, Y, and X regarding important predictors of actual COVID-19 vaccine uptake. In the case of the youngest generation, only Gen Z perceived benefits seem to matter in the decision of getting vaccinated, and these individuals seem to focus only on positive information. This finding was not underlined before in the literature in health-related outcomes. Mostly positive information processing is guiding the vaccine uptake decision in Gen X as well, with negative predictors having a low weight in total. Gen Y is an exceptional case, in which exclusively negative information processing-related variables seem to count, and all significant predictors are more relevant in the vaccine uptake refusal. These details could be useful in generation-adapted vaccination campaigns and also can serve as inspiration for cognitive bias and evolutionary perspective studies on health behavior. In the case of Gen Z and X, benefits hold the key to the decision, while in the case of Gen Y, low risks, lack of threats, and accessibility could help in the actual decision of vaccine uptake.

### Limitations and Future Direction

Beyond the new findings of the study on generational diversity in the psychological predictors of COVID-19 vaccine uptake, some limitations need to be considered too. First, the psychological factors were assessed by self-reported measures, which potentially can induce bias in the interpretation of the results. Second, the cross-sectional, one-time measurement design cannot provide information about the dynamics of the behavior. Furthermore, the recruitment of the sample was made online, by convenience sampling method, without any control or prior assessment of psychological wellbeing. All results can be interpreted only in gender-specific manner focusing on females. Further studies are needed on male samples or a more heterogeneous sample, from gender perspective. Although the total sample was adequate for analyses, the sample sizes of the three-generational cohorts were not suitable for detecting small effect sizes. The participants were recruited from different European countries, with the same ethnic background, but there might be cultural characteristics that could influence some aspects of vaccine uptake decisions. The results do not allow inferring any causality; thus future research could explore the mechanisms behind the generational diversity of COVID-19 vaccine uptake decision. For example, further studies are needed to analyze the potential moderator role of generational identity on the relationship between chronic disease and vaccination uptake, previous flu vaccine and new vaccine uptake, and also between actual disease and future vaccine uptake. Further studies are needed on possible explanations on the uplifted role of positive information (benefits related) on vaccine uptake decisions in Gen Z, and also on the highlighted generational diversity.

## Data Availability Statement

The datasets presented in this study can be found in online repositories. The names of the repository/repositories and accession number(s) can be found at: https://doi.org/10.6084/m9.figshare.19603585.

## Ethics Statement

The studies involving human participants were reviewed and approved by Ethical Committee of Babes-Bolyai University (reference number 4140/04.05.2021, Research Project: Factori psihologici predictivi ai ezitării de vaccinare împotriva COVID-19). The patients/participants provided their written informed consent to participate in this study.

## Author Contributions

All authors have contributed equally to the conception and design of the study, statistical analysis, writing, manuscript revision, and approved the submitted version.

## Funding

The publication of this article was supported by the 2022 Development Fund of the Babes-Bolyai University Cluj-Napoca, Romania.

## Conflict of Interest

The authors declare that the research was conducted in the absence of any commercial or financial relationships that could be construed as a potential conflict of interest.

## Publisher's Note

All claims expressed in this article are solely those of the authors and do not necessarily represent those of their affiliated organizations, or those of the publisher, the editors and the reviewers. Any product that may be evaluated in this article, or claim that may be made by its manufacturer, is not guaranteed or endorsed by the publisher.
